# Polyubiquitin chain-induced p62 phase separation drives autophagic cargo segregation

**DOI:** 10.1038/s41422-018-0017-7

**Published:** 2018-03-05

**Authors:** Daxiao Sun, Rongbo Wu, Jingxiang Zheng, Pilong Li, Li Yu

**Affiliations:** 1 State Key Laboratory of Membrane Biology, Tsinghua University-Peking University Joint Center for Life Sciences, School of Life Sciences, Tsinghua University, Beijing, 100084 China; 2 Beijing Advanced Innovation Center for Structural Biology, Tsinghua University-Peking University Joint Center for Life Sciences, School of Life Sciences, Tsinghua University, Beijing, 100084 China; 3 State Key Laboratory of Biomembrane and Membrane Biotechnology, Tsinghua University-Peking University Joint Center for Life Sciences, School of Life Science, Center for Nano/Micro-Mechanics and Multidisciplinary Innovation Research, Beijing, 100084 China

## Abstract

Misfolded proteins can be degraded by selective autophagy. The prevailing view is that ubiquitin-tagged misfolded proteins are assembled into aggregates by the scaffold protein p62, and the aggregates are then engulfed and degraded by autophagosomes. Here we report that p62 forms droplets in vivo which have liquid-like properties such as high sphericity, the ability to undergo fusion, and recovery after photobleaching. Recombinant p62 does not undergo phase separation in vitro; however, adding a K63 polyubiquitin chain to p62 induces p62 phase separation, which results in enrichment of high-molecular weight ubiquitin signals in p62 droplets. Mixing recombinant p62 with cytosol from p62^−/−^ cells also results in p62 phase separation in a polyubiquitination-dependent manner. Mechanistically, p62 phase separation is dependent on p62 polymerization, the interaction between p62 and ubiquitin, and the valence of the polyubiquitin chain. Moreover, p62 phase separation can be regulated by post-translational modifications such as phosphorylation. Finally, we demonstrate that disease-associated mutations in p62 can affect phase separation. We propose that polyubiquitin chain-induced p62 phase separation drives autophagic cargo concentration and segregation.

## Introduction

p62 is a common component of various cellular inclusion bodies that are often found in diseases affecting the brain and liver. These cellular inclusion bodies include Mallory–Denk bodies, intracytoplasmic hyaline bodies, and α1 antitrypsin aggregates in the liver; and Lewy bodies, neurofibrillary tangles, and huntingtin aggregates in the brain.^1, 2^ Mutations in p62 have been identified as the cause of various disease including Paget’s disease of bone (PDB) and amyotrophic lateral sclerosis (ALS).^3^ Although the precise role of p62 in these disease is not fully understood, impaired autophagy has been suggested to contribute to at least in part to the underlying pathogenic mechanism.

The roles of p62 in selective autophagy is well established. It serves as a scaffold for the formation of protein aggregates, and it acts as an autophagy receptor by linking ubiquitin-tagged protein aggregates to autophagosomes for degradation.^4^ In cultured cells, endogenous or ectopically expressed p62 forms cytoplasmic inclusion bodies (p62 bodies).^5^ p62 bodies contain polyubiquitin chains and it has been shown that K63 polyubiquitin chains are preferentially recruited into p62 bodies.^6^ Currently, p62 bodies are defined as a type of protein aggregate.^5^ However, p62 bodies are spherical and seem to grow by fusion. The spherical shape and the ability to fuse suggest that p62 bodies may not fit the classical definition of “aggregates”.

Many cellular compartments, including nucleoli, Cajal bodies, promyelocytic leukemia (PML) nuclear bodies, stress granules and P granules, are not membrane bound.^7–9^ The study of P granules in germ cells of *Caenorhabditis elegans* revealed that P granules are liquid-like and form through liquid–liquid phase separation from the cytoplasm.^10^ Subsequent studies revealed that phase separation is a common mechanism for forming non-membrane-bound compartments.^11–22^ As all these non-membrane-bound compartments have the ability to concentrate biomolecules, it was recently proposed that they should be renamed as biomolecular condensates.^23^ Biomolecular condensates substantially increase the local concentration of biomolecules, which has been proposed to have profound functional consequences, including altering the kinetics and specificity of biochemical reactions and sequestering molecules.

## Results

### P62 bodies have viscous liquid-like properties

**Fig. 1:**
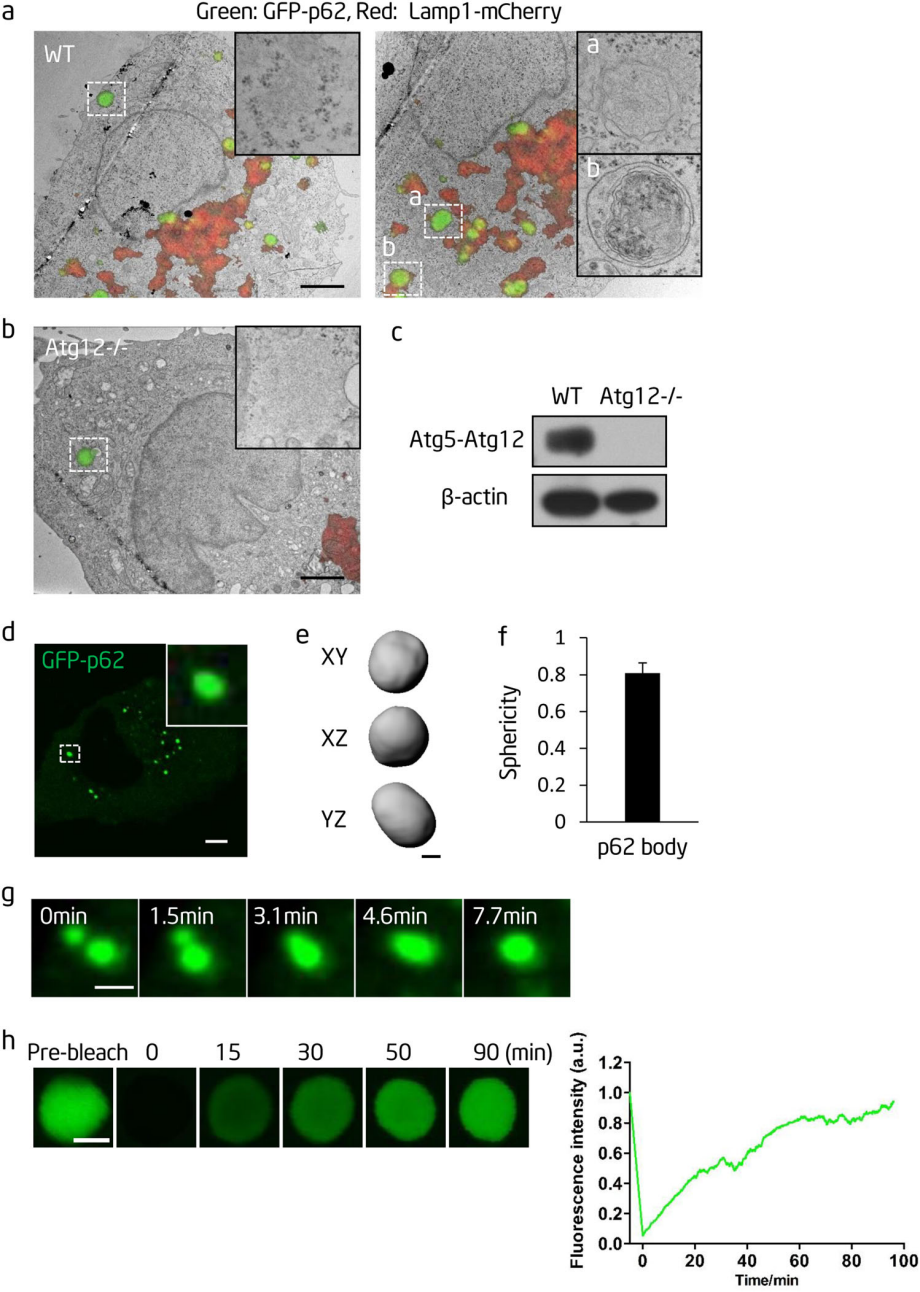
p62 forms liquid droplets in vivo. **a** Correlative light-electron microscopy (CL-EM) of NRK cells transiently transfected with GFP-p62 and Lamp1-mCherry constructs. Scale bar, 2 µm. The insert in the left panel shows a p62 body. The inserts in the right panel show p62 bodies contained within an autophagosome (upper) and an autolysosome (lower). **b** CL-EM of Atg12^−/−^ cells transiently transfected with GFP-p62 and Lamp1-mCherry constructs. Scale bar, 2 µm. The insert shows a p62 body**. c** Western blot analysis of wild-type and Atg12^−/−^ cells with the indicated antibodies. **d** GFP-labeled p62 forms p62 bodies in Atg12^−/−^ NRK cells. An enlargement of the boxed p62 body is shown in the insert. Scale bar, 5 µm. **e** Rendered 3D shapes of a p62 body. Cells were fixed with 4% PFA. The panels show the XY, XZ, and YZ planes. Scale bar, 1 µm. **f** A plot showing the sphericity of p62 bodies (*n* = 44). Error bar represents SD. **g** Fusion of p62 bodies. Scale bar, 1 µm. **h** Left panels: fluorescence intensity recovery of a p62 body after photobleaching. Scale bar, 2 µm. Right panel: quantification of fluorescence intensity recovery of a photobleached p62 body

In cultured cells, p62 puncta were observed both as non-membrane-bound p62 bodies and as p62 engulfed by autophagosomes/autolysosomes (Fig. 1a), in agreement with previous studies.^5^ Therefore, we expressed p62-GFP in autophagy-defective Atg12^−/−^ cells, in which p62 bodies cannot be taken up by autophagosomes and are maintained in the non-membrane-bound state (Fig. 1b, c). In p62-GFP-expressing Atg12^−/−^ cells, p62 bodies were spherical (Fig. 1d–f) and could undergo fusion (Fig. 1g). Furthermore, fluorescence recovery after photobleaching (FRAP) revealed that the fluorescent signal recovered after bleaching of p62 bodies (Fig. 1h), indicating that p62 can exchange among p62 bodies or between a p62 body and the surrounding cytosol. Although the recovery rate was slow, the p62 signal was almost completely restored given enough time. The slow recovery rate implies that although p62 bodies have liquid-like properties, the liquid is probably viscous. Collectively, these data suggest that p62 bodies are likely viscous liquid droplets arising from phase separation.

### Polyubiquitin chains induce p62 phase separation in vitro

**Fig. 2:**
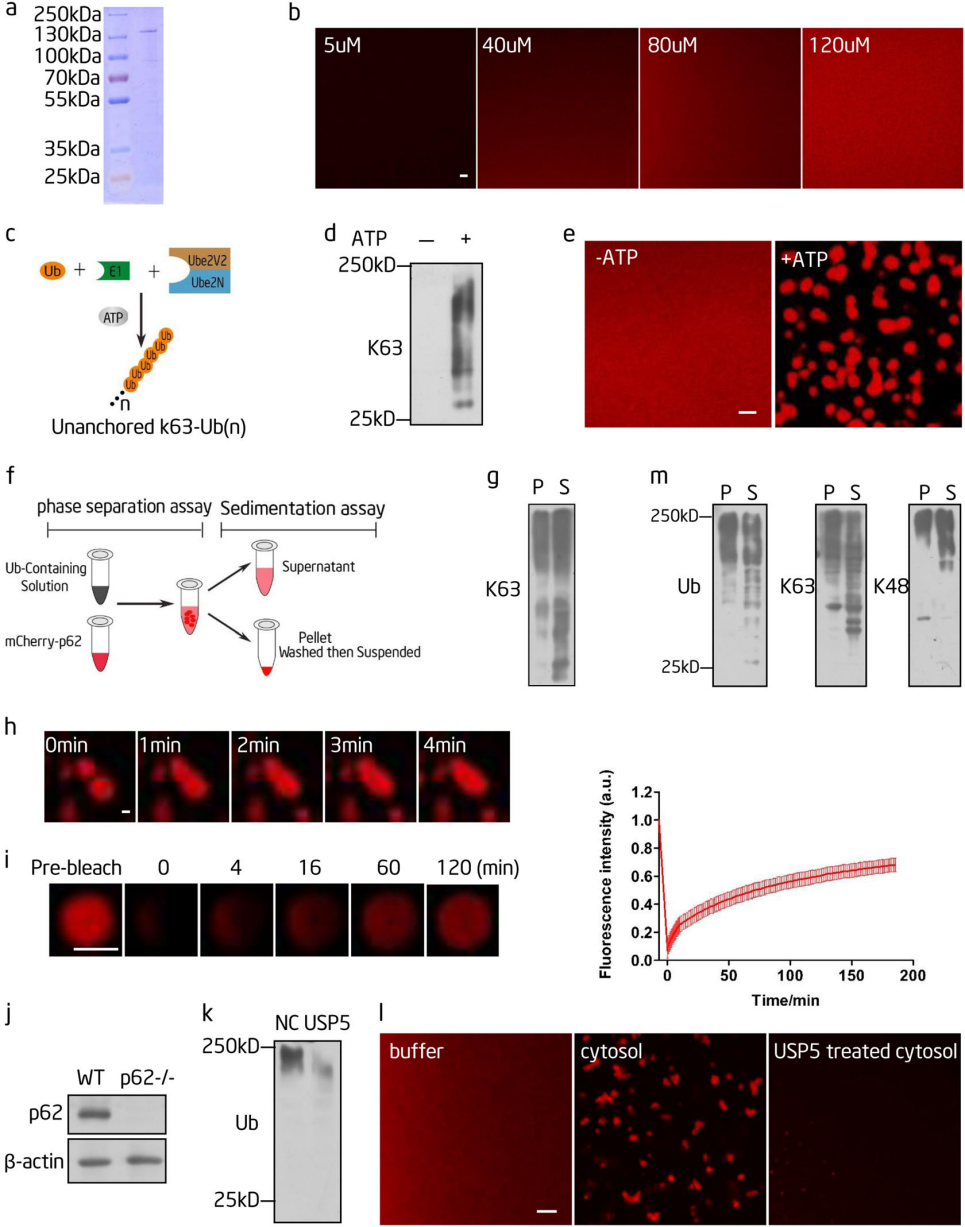
Polyubiquitin chains induce p62 phase separation in vitro. **a** The purity of purified mCherry-p62 was analyzed by Coomassie blue staining. **b** The indicated concentrations of mCherry-p62 were incubated in phase separation assay buffer and visualized by confocal microscopy. Scale bar, 5 µm. **c** Schematic diagram of the reaction to synthesize K63 polyubiquitin chains in vitro. Ub, monoubiquitin; E1, Ubiquitin-activating enzyme E1; Ube2V2/Ube2N, an E2 complex. **d** The in vitro K63 polyubiquitin chain synthesis reaction with (+) and without (-) ATP was analyzed by western blot with an anti-K63 polyubiquitin chain antibody. **e** mCherry-p62 was mixed with the in vitro-synthesized K63 polyubiquitin chain from **d** and visualized by confocal microscopy. mCherry-p62, 20 µM; monoubiquitin, 80 µM. Scale bar, 5 µm. **f** Schematic diagram of the sedimentation assay to separate the condensed liquid droplets and the supernatant. **g** The phase separation reaction from **e** was separated by centrifugation as shown in **f** and the pellet (p62 droplets) and supernatant were analyzed by western blot using an anti-K63 polyubiquitin chain antibody. S, supernatant; P, pellet. **h** Fusion of p62 droplets formed during the in vitro phase separation process in **e**. Scale bar, 1 µm. **i** Left panels: fluorescence intensity recovery of a p62 droplet formed in vitro in the presence of K63 polyubiquitin chains after half-bleaching. Scale bar, 2 µm. Right panel: quantification of fluorescence intensity recovery in the bleached region of p62 droplets (*n* = 3). **j** Western blot analysis of wild type and p62^−/−^ cells with the indicated antibodies. **k** The S150 cytosolic fraction from p62^−/−^ cells was pretreated with and without the deubiquitinating enzyme USP5 (0.025 mg/mL) for 2 h and analyzed by western blotting using an anti-ubiquitin antibody. **l** mCherry-p62 was mixed with the S150 cytosolic fraction pretreated with or without the deubiquitinating enzyme USP5 from p62^−/−^ cells and the reaction was visualized by confocal microscopy. mCherry-p62, 40 µM; cytosol, 90 mg/mL. Scale bar, 5 µm. **m** p62 droplets and supernatant from **l** were separated by centrifugation and analyzed by western blot using antibodies against ubiquitin, K63 polyubiquitin chains and K48 polyubiquitin chains. S supernatant, P pellet

Next, we tested whether p62 can undergo phase separation in vitro. No phase separation occurs in a solution containing even as much as 120 µM recombinant mCherry-p62 (Fig. 2a, b). Thus, components of the cytosol are likely required to promote the phase separation of p62. p62 can bind to polyubiquitin chains through its UBA domain, and p62 bodies contain K63 polyubiquitin chains.^4^ As it is well established that multivalent interactions drive phase separation and polyubiquitin chains are multivalent,^24^ we hypothesized that the multivalent interaction between polyubiquitin chains and p62 may promote p62 phase separation. To test this hypothesis, we generated K63 polyubiquitin chains using an in vitro ubiquitin conjugation system (Fig. 2c, d). When we mixed the recombinant mCherry-p62 and the K63 polyubiquitin conjugation reaction together, we found that phase separation occurred. In the control reaction, which did not contain adenosine triphosphate (ATP) and therefore did not form K63 polyubiquitin chains, p62 phase separation was not induced (Fig. 2e). Thus, K63 polyubiquitin chains, but not monoubiquitin or other reaction components, can induce p62 phase separation.

To determine whether polyubiquitin chains are present in p62 droplets, we separated the p62 droplets and the supernatant by centrifugation (Fig. 2f). We found that the high-molecular weight ubiquitin signal was enriched in p62 droplets (Fig. 2g). Frequently observed fusion events (Fig. 2h) and recovery of the fluorescent signal after photobleaching (Fig. 2i) indicated that these droplets were liquid droplets. Collectively, these data indicate that K63 polyubiquitin chains drive p62 phase separation. It is worth noting that the fluorescent p62 signal recovered much more slowly in vitro than in vivo, indicating that additional factors may affect the fluidity of p62 in vivo.

Next, we checked whether physiological levels of polyubiquitinated proteins are capable of causing p62 phase separation. First, we established a p62^−/−^ NRK cell line using CRISPR/Cas9 (Fig. 2j). We isolated the S150 fraction of cytosol from the p62^−/−^ cells and mixed it with recombinant mCherry-p62. As a control, we treated p62^−/−^ cytosol with UPS5, a deubiquitinating enzyme, to remove polyubiquitin chains (Fig. 2k). We found that recombinant p62 formed droplets when mixed with cytosol, but not with UPS5-treated cytosol (Fig. 2l). Similarly, we observed enrichment of higher molecular weight ubiquitin signals in p62 droplets (Fig. 2m), indicating that polyubiquitinated proteins can be segregated into p62 droplets. Further analysis showed that both K63 and K48 polyubiquitinated proteins were present in p62 droplets (Fig. 2m). Collectively, these data suggest that polyubiquitin chains are necessary to promote phase separation of p62, and in doing so, polyubiquitinated proteins are segregated into p62 droplets.

### p62 phase separation is dependent on the valence of the polyubiquitin chain

**Fig. 3:**
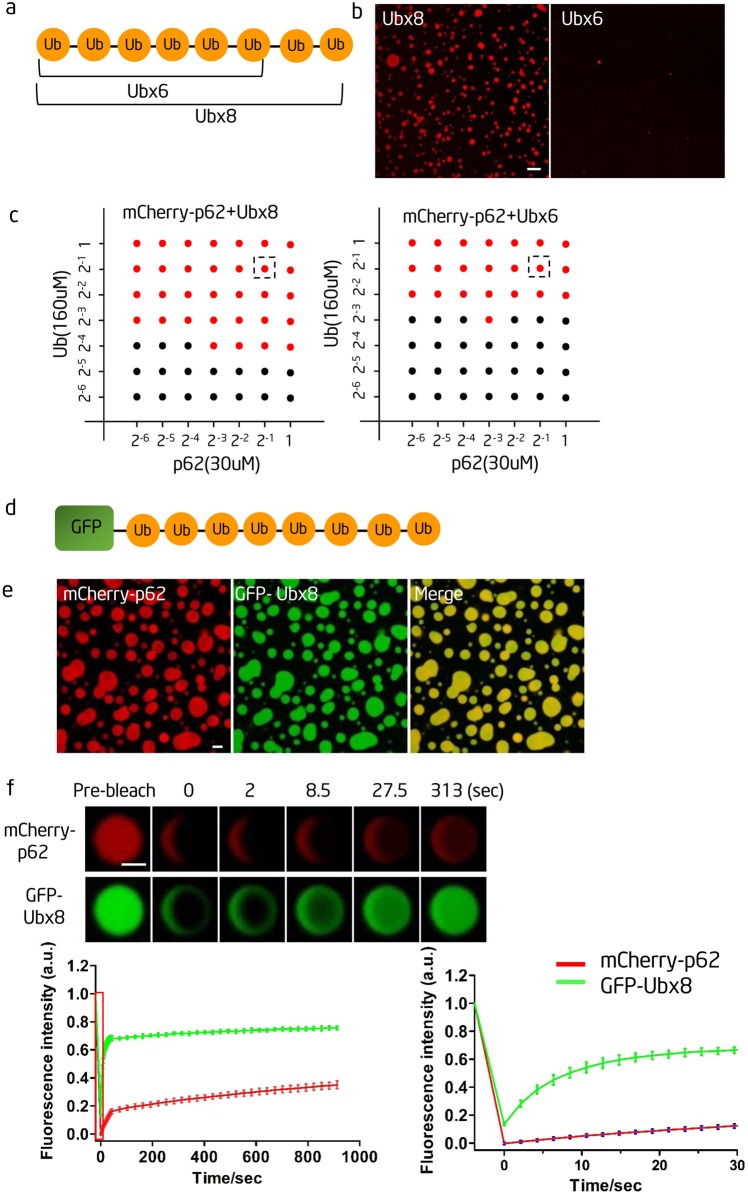
p62 phase separation is dependent on the valence of the polyubiquitin chains, and polyubiquitin chains can diffuse freely in p62 droplets. **a** Schematic diagram of linear ubiquitin chains containing 6 (Ubx6) or 8 (Ubx8) ubiquitin molecules. **b** In vitro phase separation assay of mCherry-p62 with the linear Ubx8 and Ubx6. mCherry-p62, 15 µM; Ubiquitin, 80 µM. Scale bar, 5 µm. **c** Phase diagrams of mCherry-p62 and linear polyubiquitin chains. The concentrations are in terms of the modules. The number on the *x* and *y* axes means the dilution ratio of p62 protein (30uM) and Ub (160uM).The red circles indicate phase separation, and the black circles indicate no phase separation. The boxed red circles show the concentrations used in **b**. **d** Schematic diagram of GFP-labeled linear octa-ubiquitin (GFP-Ubx8) **e** In vitro phase separation assay of mCherry-p62 with GFP-Ubx8. mCherry-p62, 20 µM; GFP-Ubx8, 5 µM. Scale bar, 5 µm. **f** Top panels: fluorescence intensity recovery of an in vitro-formed mCherry-p62 droplet in the presence of GFP-Ubx8 after photobleaching. Scale bar, 2 µm. Bottom left panel: quantification of fluorescence intensity recovery of mCherry-p62 and GFP-Ubx8 in the bleached p62 droplet. Bottom right panel: enlargement of the boxed area in the left panel (*n*=3).

Next, we investigated whether p62 phase separation is dependent on the valence of the polyubiquitin chain. As it is difficult to control the length of biochemically synthesized K63 polyubiquitin chains, we generated linear ubiquitin chains with 6 or 8 ubiquitin molecules (Ubx6 and Ubx8, respectively; Fig. 3a). We found that although both Ubx8 and Ubx6 could cause p62 phase separation, Ubx8 could induce p62 phase separation at a much lower concentration; moreover, when the concentrations of both p62 and Ub were fixed, Ubx8 induced much stronger p62 phase separation than Ubx6 (Fig. 3b, c).

To visualize the incorporation of polyubiquitin chains during p62 phase separation, we generated GFP-Ubx8 (Fig. 3d), and found that it was incorporated into droplets (Fig. 3e). FRAP experiments indicated that the GFP-Ubx8 signal recovered much faster than the p62 signal (Fig. 3f). The different recovery rates imply that p62 and GFP-Ubx8 diffuse separately and do not form a stable molecular complex; this is consistent with the idea that multivalent weak interactions often dictate phase separation.

### LC3 is recruited into p62 bodies

During autophagy, the autophagosome marker LC3 is covalently conjugated to phosphatidylethanolamine (PE) to form LC3-II, which decorates both the inner and outer membranes of autophagosomes.^25^ The un-conjugated form of LC3 is dubbed as LC3-I and the PE-conjugated LC3 is LC3-II.^25^ LC3 is the receptor for selective autophagy, and it is generally accepted that autophagosomal LC3-II can bind to proteins containing a LIR motif, thus recruiting these proteins for autophagic degradation.^26^ LC3 can directly bind to p62, and the interaction between p62 and LC3 has been proposed as the mechanism for linking p62-containing protein aggregates to the autophagy machinery.^26^

**Fig. 4:**
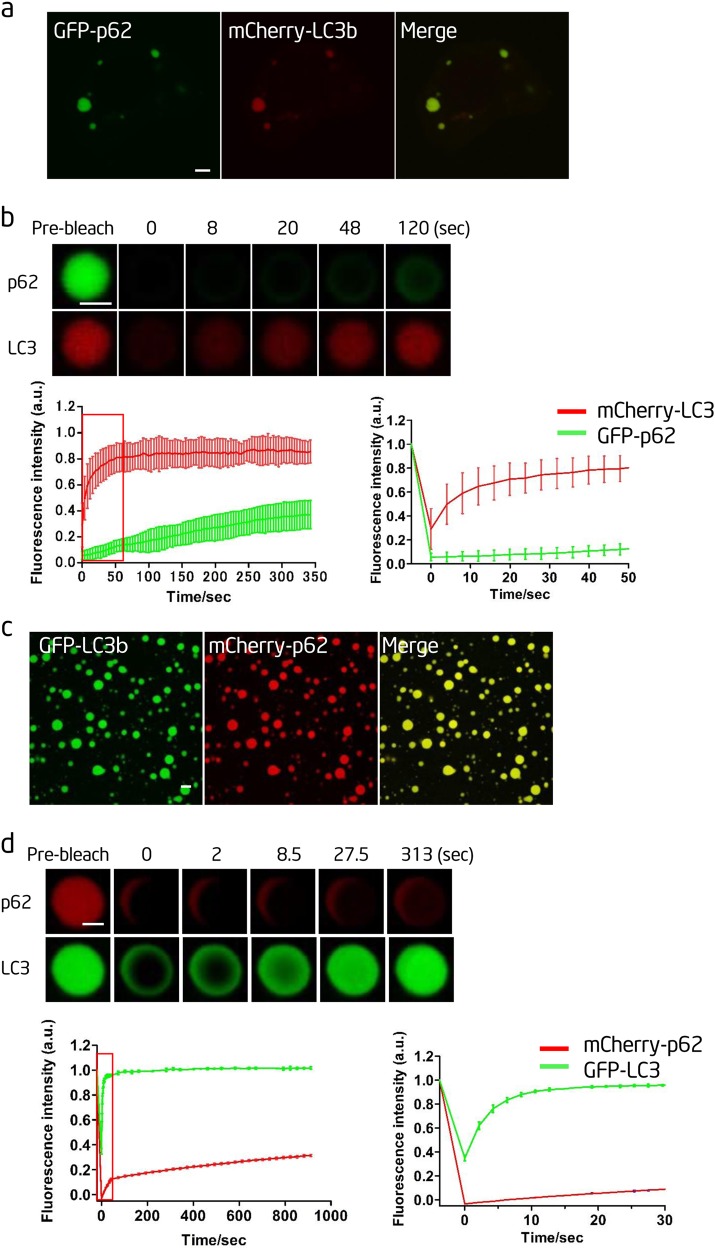
LC3 is recruited into p62 bodies. **a** Atg12^−/−^ NRK cells were transfected with mCherry-LC3b and GFP-p62 and observed by confocal microscopy. Scale bar, 5 µm. **b** Upper panels: fluorescence intensity recovery of GFP-p62 and mCherry-LC3b in a bleached p62 body. Scale bar, 2 µm. Bottom left panel: quantification of fluorescence intensity recovery of GFP-p62 and mCherry-LC3b in the bleached p62 body. Bottom right panel: enlargement of the boxed area in the left panel (*n*=5). **c** In vitro phase separation assay of mCherry-p62 with linear Ubx8. GFP-LC3b was added after the phase separation. mCherry-p62, 20 µM; Ubx8: 5 µM; GFP-LC3b, 10 µM. Scale bar, 10 µm. **d** Upper panels: fluorescence intensity recovery of GFP-LC3b and mCherry-p62 in an in vitro-formed mCherry-p62 droplet from **c** after photobleaching. Scale bar, 2 µm. Bottom left panel: quantification of fluorescence intensity recovery of GFP-LC3b and mCherry-p62 in the bleached p62 droplet. Bottom right panel: enlargement of the boxed area in the left panel (*n*=3)

We found that LC3 could colocalize with p62 in Atg12^−/−^ cells, which indicates that the un-conjugated LC3 can be recruited into p62 bodies (Fig. 4a). FRAP experiments revealed that the LC3 signal recovered much faster than the p62 signal, which indicates that LC3-I can diffuse fairly quickly inside the p62 body (Fig. 4b). Next, we tested whether LC3 could be incorporated into p62 bodies in vitro by adding the recombinant GFP-LC3 into the in vitro p62 phase separation system, we found that GFP-LC3 was incorporated into p62 droplets (Fig. 4c), and the GFP-LC3 signal recovered much faster than the p62 signal in FRAP analysis (Fig. 4d). From these data, we conclude that un-conjugated LC3 can be recruited into p62 bodies both in vivo and in vitro.

### The PB1 domain and UBA domain of p62 are required for polyubiquitin chain-induced phase separation and autophagic degradation of p62

**Fig. 5:**
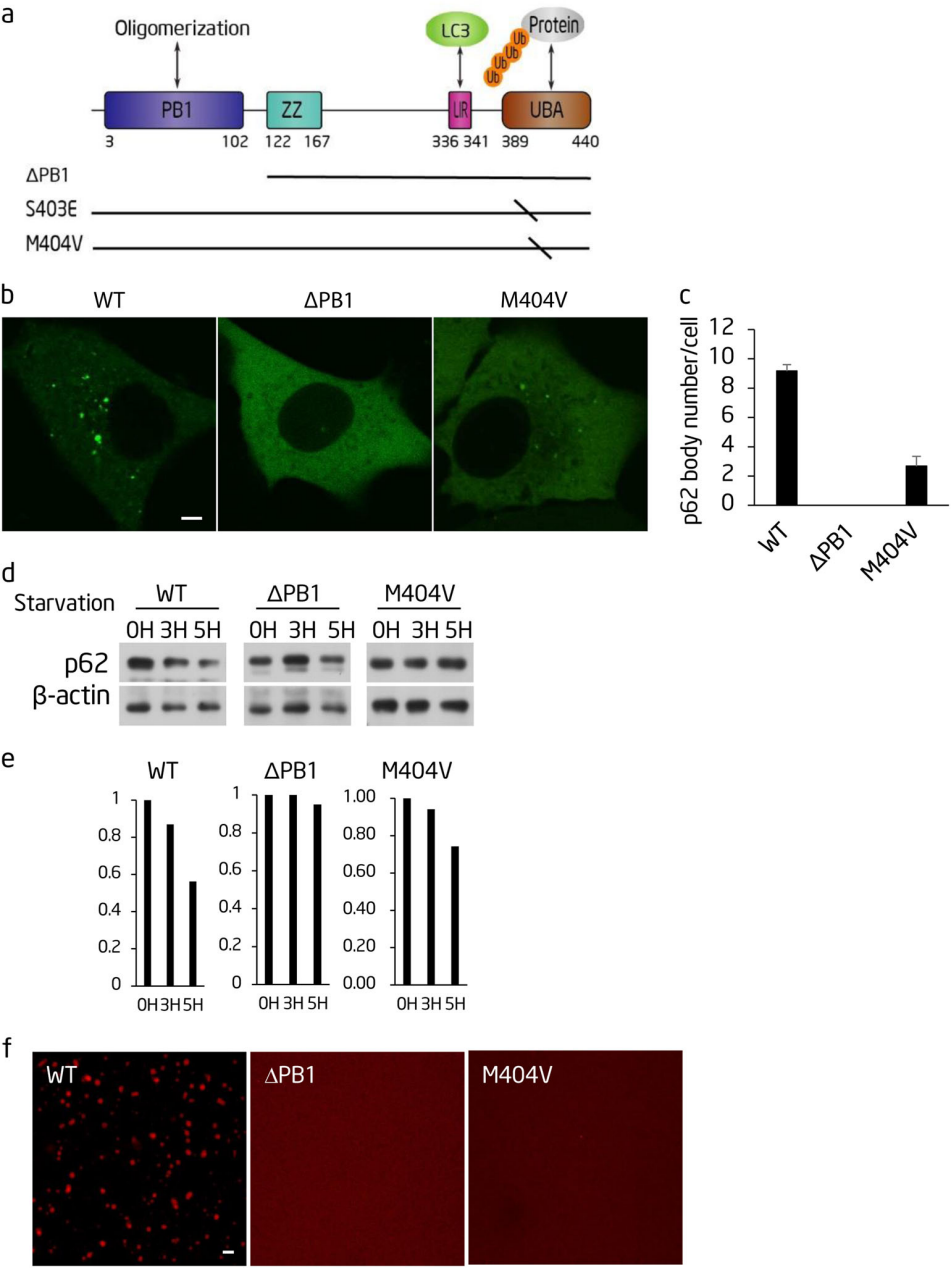
The PB1 domain and UBA domain of p62 are required for polyubiquitin chain-induced phase separation and autophagic degradation of p62. **a** Schematic diagram of the p62 protein domain structure. The location of p62 mutations is shown underneath. **b** The indicated GFP-p62 constructs were expressed in Atg12^−/−^ cells and observed by confocal microscopy. Scale bar, 5 µm. **c**. Cells from **b** were quantified for the number of p62 bodies. >45 cells from **b** were assessed blind and quantified. Error bars indicate SD (*n* = 3). **d** NRK cells transiently expressing the indicated GFP-p62 constructs were starved for the indicated time. Cell lysates were analyzed by western blot with the indicated antibodies. **e** Quantification of the indicated western blots in **d**. **f** The indicated mutated recombinant mCherry-p62 proteins were mixed with linear Ubx8 and the reaction was visualized by confocal microscopy. mCherry-p62, 5 µM; linear Ubx8, 2.5 µM. Scale bar, 5 µm

p62 has an N-terminal PB1 domain and a C-terminal ubiquitin-associated (UBA) domain (Fig. 5a). p62 is organized into flexible polymers through the PB1 domain and binds to ubiquitin through the UBA domain.^27^ In agreement with the previous literature, we found that the PB1 domain, which is required for p62 polymerization, and M404 in the UBA domain,^28^ which is essential for ubiquitin binding, are required for p62 body formation and autophagic degradation of p62 in vivo (Fig. 5b–e).

These mutations allow us to test whether polyubiquitin chain-induced p62 phase separation can be affected by p62 polymerization and by the binding affinity between p62 and K63 polyubiquitin chains. As shown in Fig. 5f, we found that polyubiquitin chain-induced p62 phase separation was markedly impaired by mCherry-p62-M404V and mCherry-p62-ΔPB1. These data suggest that p62 polymerization, as well as the interaction between polyubiquitin chains and p62, play critical roles in p62 phase separation.

### Phosphorylation of S403 promotes polyubiquitin chain-induced phase separation, p62 body formation, and autophagic degradation

**Fig. 6:**
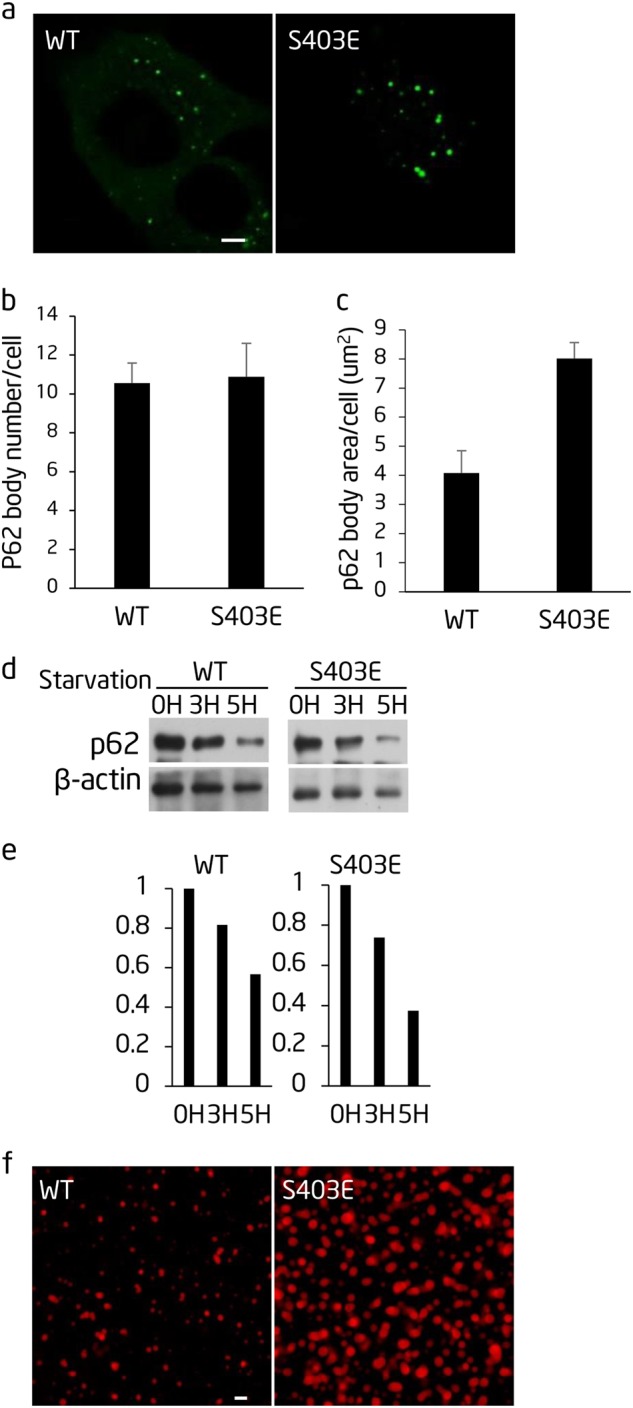
Phosphorylation of S403 promotes polyubiquitin chain-induced phase separation, p62 body formation and autophagic degradation. **a** The indicated GFP-p62 constructs were expressed in Atg12^−/−^ cells and observed by confocal microscopy. Scale bar, 5 µm. **b** Cells from **a** were quantified for the number of p62 bodies. In all, >45 cells from **a** were assessed blind and quantified. Error bars indicate SD (*n* = 3). **c** Cells from **a** were quantified for the area of p62 bodies. In total, >38 cells from **a** were assessed blind and quantified. Error bars indicate SD (*n* = 3). **d** NRK cells transiently expressing the indicated GFP-p62 constructs were starved for the indicated time. Cell lysates were analyzed by western blot with the indicated antibodies. **e** Quantification of the indicated western blots in **d**. **f** The indicated mutated recombinant mCherry-p62 proteins were mixed with linear Ubx8 and the reaction was visualized by confocal microscopy. mCherry-p62, 5 µM; linear Ubx8, 2.5 µM. Scale bar, 5 µm

p62 is subject to various post-translational modifications. It is known that phosphorylation of S403 by casein kinase 2 (CK2) increases the interaction between UBA and polyubiquitin chains.^29^ To test whether post-translational modification of p62 can affect polyubiquitin chain-induced p62 body formation and p62 phase separation, we ectopically expressed p62-S403E, which mimics S403 phosphorylation, in ATG12^−/−^ cells. We found that the number of p62 bodies was slightly higher than observed for wild type p62 (Fig. 6a, b). However, p62-S403E formed significantly larger bodies than wild-type p62 (Fig. 6a, c) and the autophagic degradation of p62-S403E was also enhanced (Fig. 6d, e). This indicates that S403 phosphorylation can promote p62 body formation and autophagic degradation. Similarly, polyubiquitin chain-induced p62 phase separation was enhanced by mCherry-p62-S403E (Fig. 6f). These results imply that p62 phase separation can be regulated by post-translational modifications such as phosphorylation.

### p62 mutations identified in PDB affect p62 body formation, autophagic degradation of p62, and p62 phase separation

**Fig. 7:**
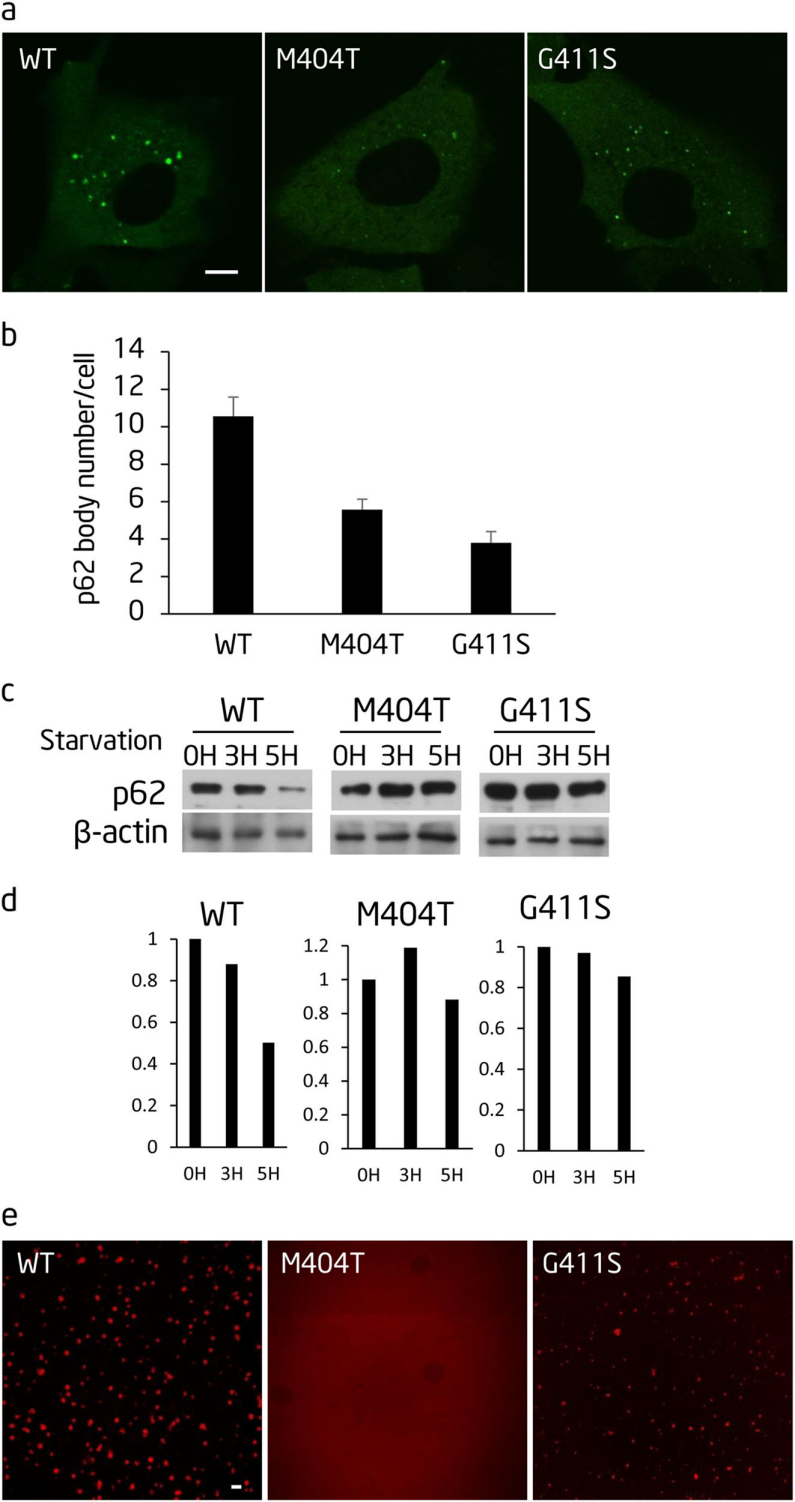
Two p62 mutations identified in Paget’s disease of bone (PDB) affect p62 body formation, autophagic degradation of p62, and p62 phase separation. **a** The indicated GFP-p62 constructs were expressed in Atg12^−/−^ cells and observed by confocal microscopy. Scale bar, 5 µm. **b** Cells from **a** were quantified for the number of p62 bodies. In all, >45 cells from **a** were assessed blind and quantified. Error bars indicate SD (*n* = 3). **c** NRK cells transiently expressing the indicated GFP-p62 constructs were starved for the indicated time. Cell lysates were analyzed by western blot with the indicated antibodies. **d** Quantification of the indicated western blots in **c**. **e** The indicated mutated recombinant mCherry-p62 proteins were mixed with linear Ubx8 and the reaction was visualized by confocal microscopy. mCherry-p62, 5 µM; linear Ubx8, 2.5 µM. Scale bar, 5 µm

Mutations in p62 have been identified as the cause of PDB. So far, all the mutations identified in PDB are located in the UBA domain. The fact that M404V is associated with PDB promote us to investigate whether PDB mutations affect phase separation of p62; for this purpose, we generated p62-GFP with two PDB mutations, M404T and G411S.^3^ We then tested the ability of the mutant proteins to form p62 bodies and to undergo starvation-induced degradation in p62^−/−^ cells. We found that both of these mutations affected p62 body formation and autophagic degradation of p62 (Fig. 7a-d). Moreover, polyubiquitin chain-induced p62 phase separation was markedly impaired by these mutations (Fig. 7e).

## Discussion

**Fig. 8:**
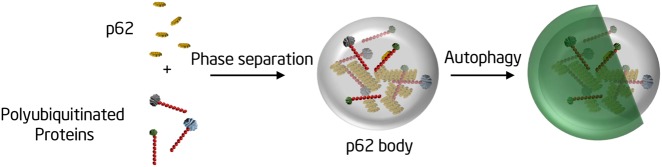
Model for the formation of p62 bodies and their role in selective autophagy

For effective degradation by selective autophagy, cargo proteins must be concentrated and segregated into a physical entity, so that they can be surrounded and taken up by the autophagosomal membrane. The prevailing view is that autophagic cargo proteins are concentrated and segregated by forming aggregates. Based on data presented here, we propose a model in which autophagic cargo proteins are concentrated and segregated by polyubiquitin chain-induced phase separation of p62 (Fig. 8). In this model, the polyubiquitin chain is not only a tag for marking proteins destined for autophagic degradation, but also acts as an activation signal for triggering cargo segregation.

The distinction between protein aggregates and phase separation droplets may have significant functional consequences for selective autophagy and beyond. Proteins in aggregates are generally misfolded and lack mobility, and it is unlikely that biochemical reactions can occur inside aggregates. In contrast, proteins in droplets can keep their conformation and activity, and can diffuse within the droplet and into the surrounding environment. Thus, our model suggests that besides concentrating and segregating cargo, p62 droplets may also serve as an organizing center for signaling cascades that initiate selective autophagy. In sum, our data provide a fundamentally different perspective on autophagic degradation of aggregates and aggregate formation in general.

LC3 can bind many proteins containing a LIR motif. In this study, we demonstrate that un-conjugated LC3 can be recruited into p62 droplets in vitro and p62 bodies in vivo. This observation raises the interesting possibility that LIR motif-containing proteins can be selectively recruited into p62 bodies by LC3, which may contribute to the selectivity of p62-mediated aggrephagy.

In this article, we show that all the mutant p62 proteins with impaired phase separation are also impaired in their ability to form p62 bodies and undergo autophagic degradation in vivo. Our data suggest that phase separation of p62 is required for p62 body formation and autophagic degradation of p62. Besides phase separation, other mechanisms can also contribute to p62 body formation in vivo. Therefore, our results do not imply that all p62 mutants, which are defective in p62 body formation and autophagic degradation will also be defective in phase separation.

p62 point mutations have been reported in PDB and ALS. In all, 25–50% of familial PDB patients have a mutation in p62, and most of the mutations are located in the UBA domain. At this point, although increase of nuclear factor-κB activity and autophagy dysfunction have been linked to these mutations, the precise mechanisms by which these mutations cause disease are not fully understood. Our data suggest that impairment of phase separation may be the common mechanism underlying the diverse biological processes affected by these mutations.

## Materials and methods

### Constructs

The GFP-p62 constructs were generated by cloning human p62 into pEFGP-C3. The MBP-mCherry-p62 constructs were generated by cloning human p62 into His6-MBP-Tev-mCherry vector. The M404V, M404T, G411S, S403E, and ΔPB1 mutants were generated by PCR-based mutagenesis.

### Protein expression and purification

All proteins were expressed in *Escherichia coli* BL21 Rosetta (DE3) cells. For mCherry-p62-WT/ΔPB1/S403E/M404V/M404T/G411S, cells were re-suspended in lysis buffer (150 mM NaCl, 40 mM Tris-HCl pH 7.4, 10% glycerol, 4 mM Dithiothreitol (DTT), 1 mM EDTA, protease inhibitor cocktail). mCherry-p62-WT/S403E/M404V/M404T/G411S were purified through a MBPTrap HP column. p62 (ΔPB1) was first isolated through a Ni-NTA column and further purified using a Hitrap Q HP column (GE Healthcare). Proteins were concentrated by centrifugal filtration (100 K MWCO) and then purified using a Superose 6 10/300 gel filtration column (GE Healthcare) equilibrated in 150 mM NaCl, 40 mM Tris-HCl pH 7.4, 10% glycerol, 1 mM DTT. The proteins were flash-frozen and stored at −80 °C.

For His6-UBx6/His6-UBx8, His6-GFP-Ubx8, and His6-Ube2V2/His6-Ube2N, bacteria were grown in lysogeny broth (LB) at 37 °C and induced at OD_600_ ~0.8 with 0.5 mM IPTG at 18 °C for 16 h. Cells were re-suspended in 150 mM NaCl, 40 mM Tris-HCl pH 7.4, 10% glycerol, 1 mM DTT, 1 mM EDTA, 1 mM phenylmethylsulfonyl fluoride (PMSF), and disrupted with sonication on ice, followed by centrifugation at 20,000 rpm for 1 h at 4 °C. All these proteins were isolated first through a Ni-NTA column. His6-Ube2V2/His6-Ube2N and His6-GFP-Ubx8 were further purified using a Hitrap Q HP column. Solution of His6-Ubx6 and His6-Ubx8 were acidified using acetic acid, then applied to a Hitrap SP HP column, and finally eluted with a linear gradient of NaCl (0–1 M) in 50 mM ammonium acetate pH 4.5. All proteins were further purified by gel filtration. The gel filtration columns (superdex 75 10/300 for His-Ub6 and His6-Ube2V2/His6-Ube2N; superdex 200 10/300 for His6-Ubx8 and His6-GFP-Ubx8) were equilibrated in 150 mM NaCl, 40 mM Tris-HCl pH 7.4, 10% glycerol, 1 mM DTT. The proteins were flash-frozen and stored at −80 °C.

For His6-MBP-SUMO-Ub, the recombinant protein was purified on a Ni-NTA column and a Hitrap Q HP column, and the His6-MBP-SUMO tag was cleaved by ULP1 enzyme overnight at 4 °C. The protein was then passed through a Ni-NTA column and a MBPTrap HP column. Unbound proteins were applied to superdex 75. After cleavage, the mono-Ub containing an N-terminal linker of three residues (Serine-Methionine-Glycine) was used for the synthesis of K63 polyubiquitin chains.

His6-UBE1 was expressed in insect cells, and purified on a Ni-NTA column and a Hitrap Q HP column.

An additional ultra-centrifugation step (12,000 rpm, 10 min) was applied to remove potential aggregates of all protein samples prior to droplet assembly experiments. The concentrations of all the proteins were measured from the absorbance at 280 nm using a Nanodrop and the extinction coefficients were obtained using the primary sequences of the proteins using ExPASy ProtParam (http://web.expasy.org/protparam/)

### Synthesis of K63 polyubiquitin chains

The pre-initiation solution included 0.1 µM E1, 20 µM His6-Ube2V2/His6-Ube2N, 640 µM mono-Ub, 5 mM MgCl_2_, 50 mM NaCl, 50 mM Tris-HCl pH 7.6, 0.2 mM DTT. The conjugation reaction was initiated by adding 4 mM ATP and then incubating at 37 °C for 4 h. Solution without ATP was processed as the control.

### Phase separation assay

Phase separation assay was performed in 150 mM NaCl, 40 mM Tris-HCl pH 7.4, 10% glycerol, 1 mM DTT, with the indicated protein concentrations. TEV protease was used to remove the N-terminal His-MBP tag of mCherry-p62 proteins before reaction. For FRAP and time-lapse imaging experiments, phase separation assay was carried out on glass-bottomed 35 mm dishes (In Vitro Scientific), which were coated with 3% bovine serum albumin for 15 min and then washed with Milli Q H_2_O three times.

### Sedimentation assay

After phase separation assay, p62 droplets were spun down at 12,000 *g* for 5 min. The pellet was washed with phase separation buffer once gently. The amount of the ubiquitin (Cayman; clone FK1), K63 polyubiquitin (Abcam; cat: ab179434), K48 polyubiquitin (Abcam; cat: ab140601) were analyzed by western blot.

### Cell culture, transfection, and imaging

NRK cells were cultured in Dulbecco’s modified Eagle’s medium (Life Technologies) supplemented with 10% fetal bovine serum and 50 µg/mL penicillin and streptomycin in a 5% CO_2_ incubator. For starvation, cells were washed twice with phosphate-buffered saline and incubated in starvation medium (Life Technologies; cat 11960) for the indicated time. Cells were transfected with a total of 2 µg DNA by Amaxa nucleofection using solution T and program X-001. Living cell images were acquired using a confocal microscope (FV-1000; Olympus) that was equipped with Uplansapo 60 × /1.35 oil immersion and WHN 20 × / 22 lenses. Imaging medium was Immersion Oil Type-E (Olympus). Image acquisition and processing were performed using FV10-ASW 3.1 software (Applied Precision).

### Three-dimensional (3D) rendering and sphericity measurement

NRK cells transiently transfected with GFP-p62 were fixed with 4% paraformaldehyde (PFA). Images were acquired using a confocal microscope (NIKON A1) with a 60× oil immersion lens. The step size was 0.125 µm. 3D rendering was performed using Imaris software and calibrated with spherical beads. Sphericity was calculated using NIKON A1 analysis software.

### Spinning disk microscopy

To observe the fusion events of p62 bodies in vivo, living NRK cells were starved with starvation medium and imaged using an UltraView Vox Spinning Disc Microscopes with a 60× oil immersion objective (Olympus).

### Fluorescence recovery after photobleaching

FRAP experiments were performed on a NIKON A1 microscope with a 60× oil immersion objective. p62 bodies (in vivo) or p62 droplets (in vitro) were bleached for 3 s using a laser intensity of 70% at 480 nm (for GFP) or 561 nm (for mCherry). Recovery was recorded for the indicated time. The fluorescence intensity of the photobleached area was normalized to the intensity of the unbleached area.

### Correlative confocal and electron microscopy

Live-cell dishes with photo-etched gridded coverslips were used to culture NRK or Atg12^−/−^ cells. Cells were fixed with 4% PFA first and then a confocal microscope was used to collect 1–2 bright field and confocal images to document the arrangement of the cells at different magnifications. After that, the cells were fixed with 2.5% glutaraldehyde (GA) for 2 h at room temperature. Samples were then dehydrated with a graded ethanol series (50%, 70%, 90%, 95%, and 100%) for 8 min each. Samples were infiltrated with and embedded in SPON12 resin. After polymerizing for 48 h at 60 °C, 70-nm-thick ultrathin sections were cut using a diamond knife, and then picked up with Formvar-coated copper grids (100 mesh). The sections were double stained with uranyl acetate and lead citrate. After air drying, samples were examined with a transmission electron microscope H-7650 at an acceleration voltage of 80 kV.
